# Prevalence of acute pain crisis in patients with cancer in Specialist Palliative Care Clinic: An observational study

**DOI:** 10.1186/s12904-026-02013-3

**Published:** 2026-02-21

**Authors:** Shruti Kamble, Raghu S. Thota, Shamali Poojary, Jayita Deodhar, Varun T. M., Isha J Shah, Ajila Ajith

**Affiliations:** Department of Palliative Medicine, Tata Memorial Hospital, Homi Bhabha National Institute, Mumbai, 400012 India

**Keywords:** Acute pain crisis, Cancer, Palliative care

## Abstract

**Background:**

To evaluate the prevalence of acute pain crisis among cancer patients presenting to an outpatient specialist palliative care clinic.

**Methods:**

This observational study was conducted over six months at a tertiary cancer hospital in India utilizing the ESAS-r and Distress Thermometer tools. Eligible participants were patients aged 18 years or older who attended the Palliative Medicine outpatient clinic with a pain score of 7 or above on the Numerical Rating Scale and a distress score of 4 or higher on the Distress Thermometer.

**Results:**

Of the 5,570 patients screened for eligibility, 95 presented with an acute pain crisis, yielding a prevalence of 1.7% (95% CI: 1.38–2.08). The median age of the patients was 50 years (IQR: 40–57), with 54% females and 46% males. The most common cancer sites were the head and neck (20%), followed by gynecological (13.7%), and thoracic/breast/hepatobiliary (10.5%) cancers.

The most frequent pain quality descriptors were radiating pain (59%), shooting (49%), electrical or throbbing (44%), pricking (43%), dull aching (38%), burning (27%), tenderness (20%), cramping (17%), and unknown types (2%). The median distress score reported on the Distress Thermometer was 8 (IQR: 6–9).

Intravenous fentanyl was the most commonly used analgesic for managing pain crisis, with a median dose of 20 mcg (IQR: 20). The median NRS pain scores were 9 (IQR: 8–10) at 0 minutes, 6 (IQR: 4–7) at 15 minutes, and 3 (IQR: 2–4) at 30 minutes. The reductions in NRS scores at 15 and 30 minutes were 3 and 6 points, respectively.

**Conclusion:**

To the best of our knowledge, this is the first study aimed at evaluating the prevalence of pain crisis in an outpatient specialist palliative care clinic. Managing an acute pain crisis in such a setting requires a careful balance of timely interventions, appropriate medication use, and strong communication among patients, caregivers, and palliative care physicians. The causes of acute pain crisis were not explored in this study, which represents a potential area for future research.

**Trial registration:**

Clinical Trials Registry India (CTRI) Reg no. CTRI/2024/02/062517 [Registered on 12/02/2024].

## Background and rationale

Pain is a distressing symptom that evokes both sensory and emotional suffering, significantly impacting patients as well as their caregivers. In recognition of the complexity of pain experiences, the International Association for the Study of Pain (IASP) revised its definition in 2020 to *“An unpleasant sensory and emotional experience associated with*,* or resembling that associated with actual or potential tissue damage.”* This updated definition underscores the multidimensional nature of pain and emphasizes the need for comprehensive approaches to its assessment and management [1]. 

In palliative care, the inherently subjective experience of pain poses challenges for objective evaluation. The emotional, psychological, and existential components further complicate its characterization, necessitating individualized and nuanced clinical approaches. Accurate pain assessment is essential for understanding its etiology—whether related to the underlying malignancy, treatment modalities, or coexisting conditions—and for distinguishing between somatic, visceral, neuropathic, or mixed pain types, including the presence of breakthrough pain. These distinctions are fundamental to guiding clinical decision-making and optimizing analgesic strategies [2]. 

An acute pain crisis represents a particularly severe manifestation of pain. Moryl et al. (2008), in *Clinicians Corner*,* JAMA*, defined it as an event involving intense, uncontrolled pain that causes significant distress to both patients and their families [3]. Despite the clinical importance of this phenomenon, standardized definitions, diagnostic criteria, and treatment protocols for pain crisis are lacking, which hampers timely and effective management.

Acute pain crisis is distinguished from breakthrough cancer pain by the presence of poorly controlled or worsening baseline pain.

A recent systematic review and meta-analysis by Snijders et al. (2023), encompassing 444 studies published between January 2014 and December 2021, reported a pooled prevalence of overall pain in cancer patients of 44.5% (95% CI: 41.1–47.9) and moderate to severe pain of 30.6% (95% CI: 26.9–34.4). Among those receiving palliative care, the overall prevalence of pain increased to 54.3% (95% CI: 42.0–66.6), with 39.1% (95% CI: 30.0–48.1) experiencing moderate to severe pain. The highest prevalence was observed among patients who were no longer eligible for disease-modifying treatment, with 55.2% (95% CI: 39.2–71.3) experiencing pain and 43.3% (95% CI: 19.7–66.9) reporting moderate to severe pain [4]. 

Despite these alarming figures, the literature on the prevalence, characterization, and management of acute pain crisis remains sparse in outpatient setting. Furthermore, existing clinical guidelines advocate opioid use and emergency hospitalization for patients in pain crisis [5–7]; however, there is limited consensus and empirical evidence to guide standardized care.

This study aims to systematically examine the prevalence and management strategies of acute pain crisis in palliative care settings, to generate evidence to inform clinical best practices and guide policy development.

## Methods

### Study design

This was a prospective observational study conducted in the outpatient clinic of the Department of Palliative Medicine at a tertiary cancer hospital in India. The study was designed and conducted in accordance with the ethical principles outlined in the Declaration of Helsinki. Only patients who met the inclusion criteria and provided informed consent to participate were enrolled. The study protocol was reviewed and approved by the Institutional Ethics Committee of the hospital and was prospectively registered with the Clinical Trials Registry of India (CTRI Reg. No. CTRI/2024/02/062517; Registered on: 12/02/2024).

Patients with advanced cancer receiving palliative intent treatment were recruited from the outpatient palliative medicine clinic on the basis of predefined eligibility criteria. The inclusion criteria included patients aged 18 years or older who presented to the clinic with a pain score of ≥ 7 on the NRS and a distress score of ≥ 4 on the Distress Thermometer. Additionally, patients referred for early palliative care or those with no cancer-directed treatment were also considered eligible for inclusion. The exclusion criteria included a history of known allergies or adverse reactions to analgesics, as well as cognitive impairment that could interfere with assessment or communication.

### Outcomes and measures

The primary objective was to determine the prevalence of acute pain crisis in patients with cancer presenting at outpatient specialist palliative care clinics.

The secondary objectives of this study included the characterization of pain using pain quality descriptors. The study also assessed the level of distress experienced by patients during these episodes, documented the types, doses, and routes of analgesics administered for their management; and evaluated the effectiveness of these interventions by measuring changes in pain scores at 15 and 30 min after administration.

The severity of pain was recorded via a standard numerical rating scale. Symptom burden was assessed via the Edmonton Symptom Assessment System–Revised (ESAS-r) [8], and distress levels were measured via the Distress Thermometer [9]. Demographic details were collected from the electronic medical records (EMRs). The evaluation of pain quality descriptors was assessed via a binary response format (yes/no) for the presence of specific characteristics such as burning, pricking, shooting, electrical/shock-like, throbbing, radiating, numbing, dull aching, cramping, and tenderness.

### Sample size and statistical considerations

Since there are no historical or previous data available to estimate the prevalence of acute pain crisis in patients with cancer presenting in an outpatient specialist palliative care clinic, we can consider this study exploratory in nature. Importantly, the sample size in exploratory studies is not based on statistical power calculations. To assess the significance of the change in pain scores, paired t tests or Wilcoxon signed-rank tests were used, and the results are presented as the means or medians of the changes in pain scores, along with the corresponding p values. A *p value* of less than 0.05 was considered statistically significant. The statistical analysis was conducted via SPSS version 25 and R Studio.

## Results

**Fig. 1:**
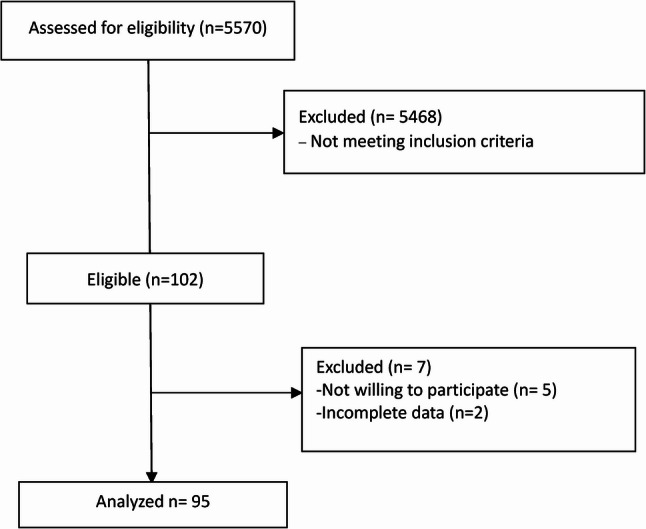
Consort flow chart

A total of 5,570 patients were screened for eligibility, and 95 were enrolled as per the inclusion criteria (Fig. 1). The prevalence was found to be 1.71% (95% CI: 1.38–2.08), with a median distress score of 8 [interquartile range (IQR): [6–9]. The median age of the patients was 50 years (IQR: 40–57). The most common primary cancer site was the head and neck (20%), followed by gynecological (13.7%) and thoracic/breast/hepatobiliary (10.5%) cancers. Hematolymphoid malignancies were the least common (1.1%) (Table 1).

**Table 1:** Patient characteristics

	*n* (%)
Total Patients	95
Gender Distribution	
Male	44 (46%)
Female	51 (54%)
Age	
Median (IQR)	50 (40–57)
Comorbidity	
Hypertension	8 (8.4)
Diabetes Mellitus	7 (7.3)
Hypothyroid	7 (7.3)
Coronary Artery Disease	1 (1)
Cancer Diagnosis	
Breast	10 (10.5)
Bone and Soft Tissue	9 (9.5)
Carcinoma of Unknown Primary	3(3.2)
Thoracic	10 (10.5)
Renal	4 (4.2)
Melanoma	3 (3.2)
Hepatobiliary	10 (10.5)
Hematolymphoid	1 (1.1)
Head and Neck	19 (20)
Gynecology	13 (13.7)
Gastrointestinal	9 (9.5)
Urology	3 (4.2)
Metastatic	
Yes	51(53)
No	44(46)
Undergoing Disease Directed Treatment	
Yes	33(35)
No	61 (64)
Underevaluation	1 (1)
Opioid Naive	33 (35)
Opioid Tolerant	62 (65)
Compliant to analgesics	66 (80)

A majority of patients (67%, *n* = 62) were opioid tolerant, whereas 33% (*n* = 31) were opioid naïve. The US Food and Drug Administration (FDA) defines a patient as opioid tolerant if for at least 1 week he or she has been receiving oral morphine 60 mg/day; transdermal fentanyl 25 mcg/hour; oral oxycodone 30 mg/day; oral hydromorphone 8 mg/day; oral oxymorphone 25 mg/day; or an equianalgesic dose of any other opioid. The mean morphine milligram equivalent (MME) per day was 46 mg (SD: 36), with a median of 50 mg (IQR: 15–60).

Paracetamol was the most frequently prescribed oral analgesic (60%), followed by gabapentin (21%), pregabalin (17%) and non-steroidal anti-inflammatory drugs (5%). Notably, 20% of patients were not on any ongoing oral analgesics (Table 2).

**Table 2:** Analgesics used in opioid Naive and opioid tolerant participants

Sr.No	Opioid Naive	Opioid Tolerant
1.	Paracetamol	Tramadol
2.	Gabapentin	Morphine
3.	Pregabalin	Buprenorphine
4.	Amitryptiline	Fentanyl
5.	Nortryptiline	
6.	Dexamethasone	
7.	Etoricoxib	
8.	Diclofenac	
9.	Dicyclomine	

**Fig. 2:**
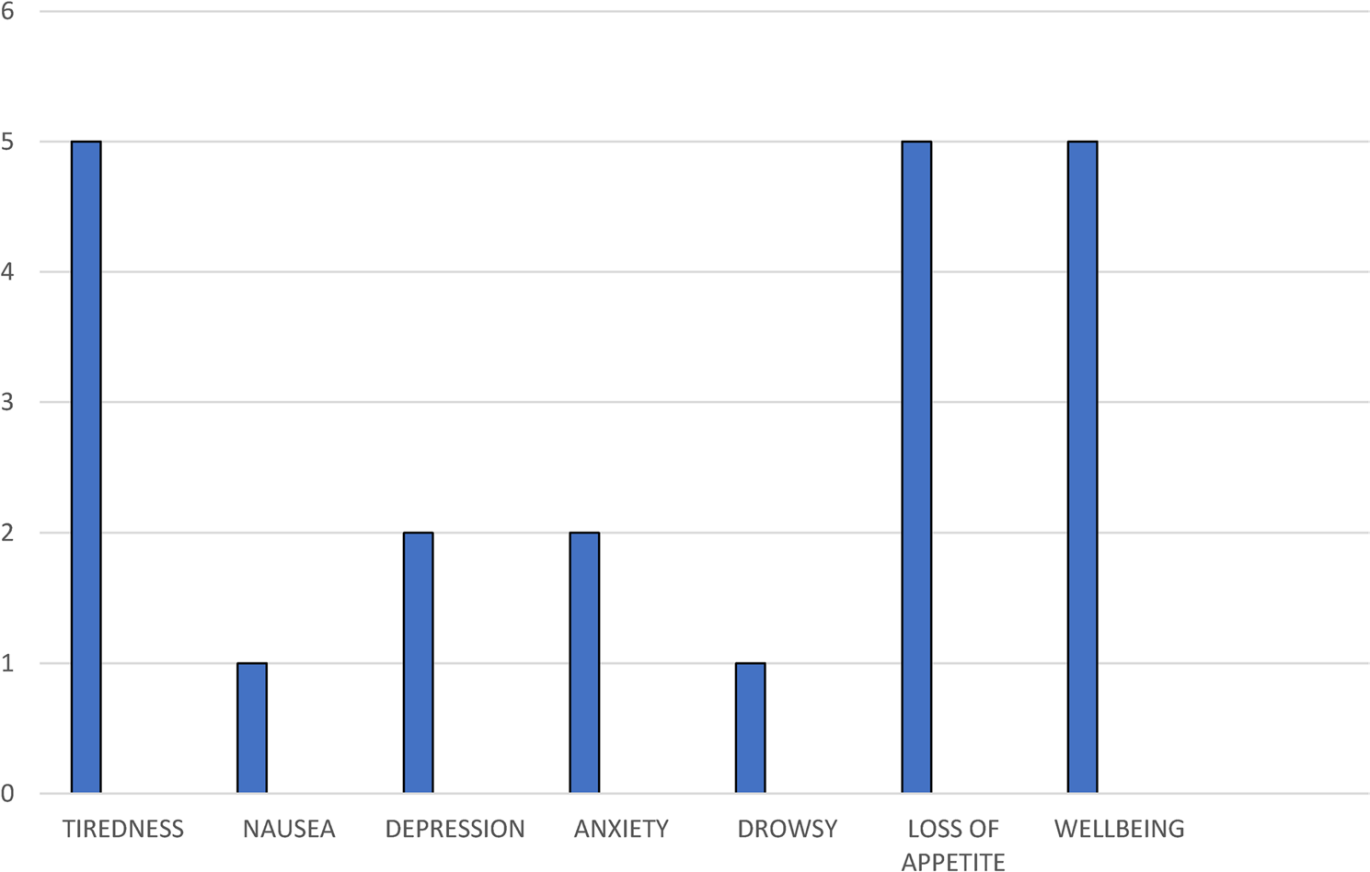
Median ESAS-r scores

According to the Edmonton Symptom Assessment Scale–Revised (ESAS-r), the highest-rated symptoms were tiredness, loss of appetite, and poor overall well-being, with anxiety and depression also commonly reported (Fig. 2). The mean score on the Distress Thermometer was 7.24 (SD: 2.19), with a median of 8 (IQR: 6–9).

The pain quality descriptors most frequently reported included radiating (59%), shooting (49%), electrical and throbbing (44% each), pricking (43%), dull aching (38%), burning (27%), tenderness (20%), and cramping (17%). Only 2% reported the pain quality as unknown.

**Fig. 3:**
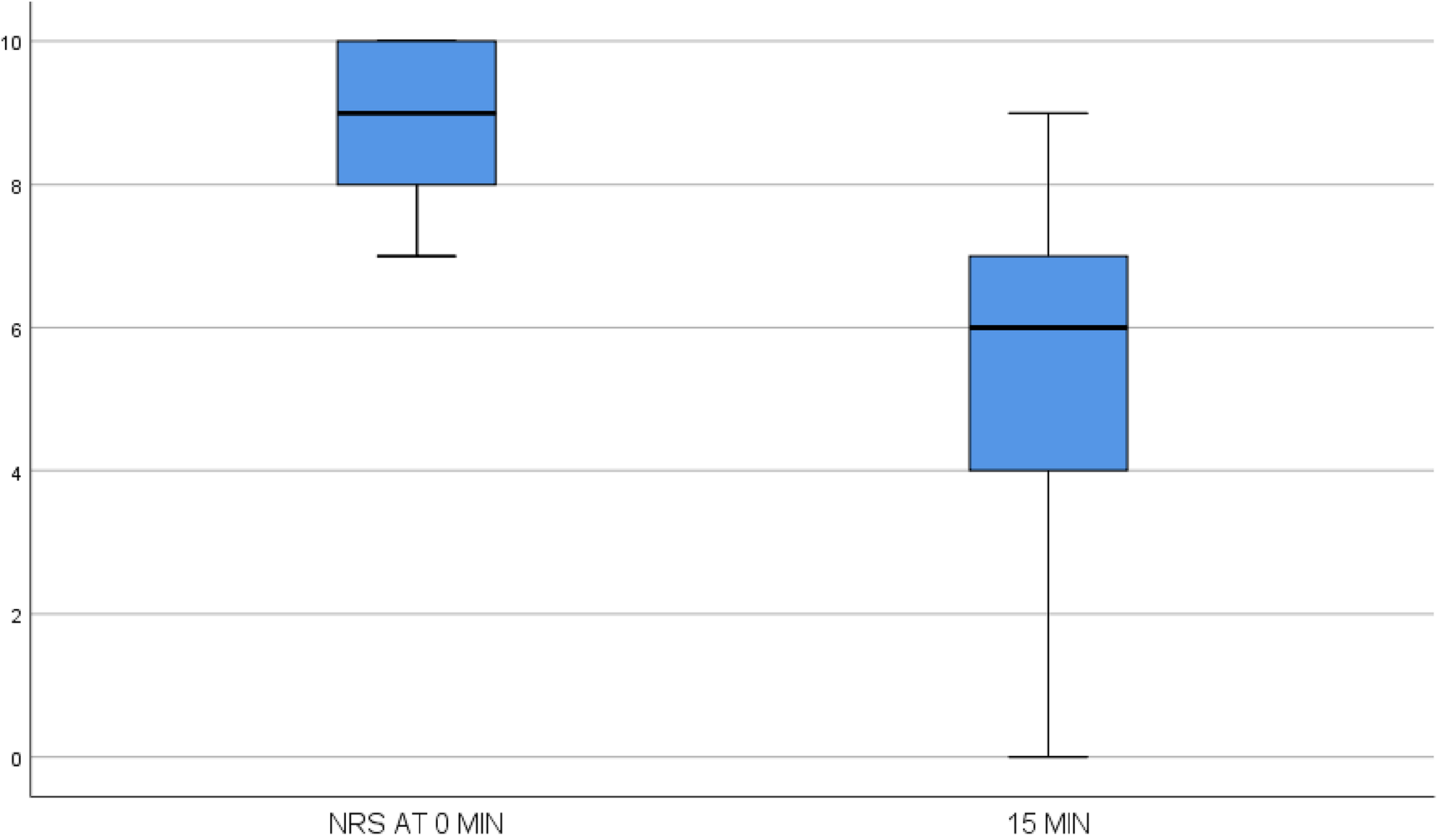
Changes in the NRS score at 15 min

**Fig. 4:**
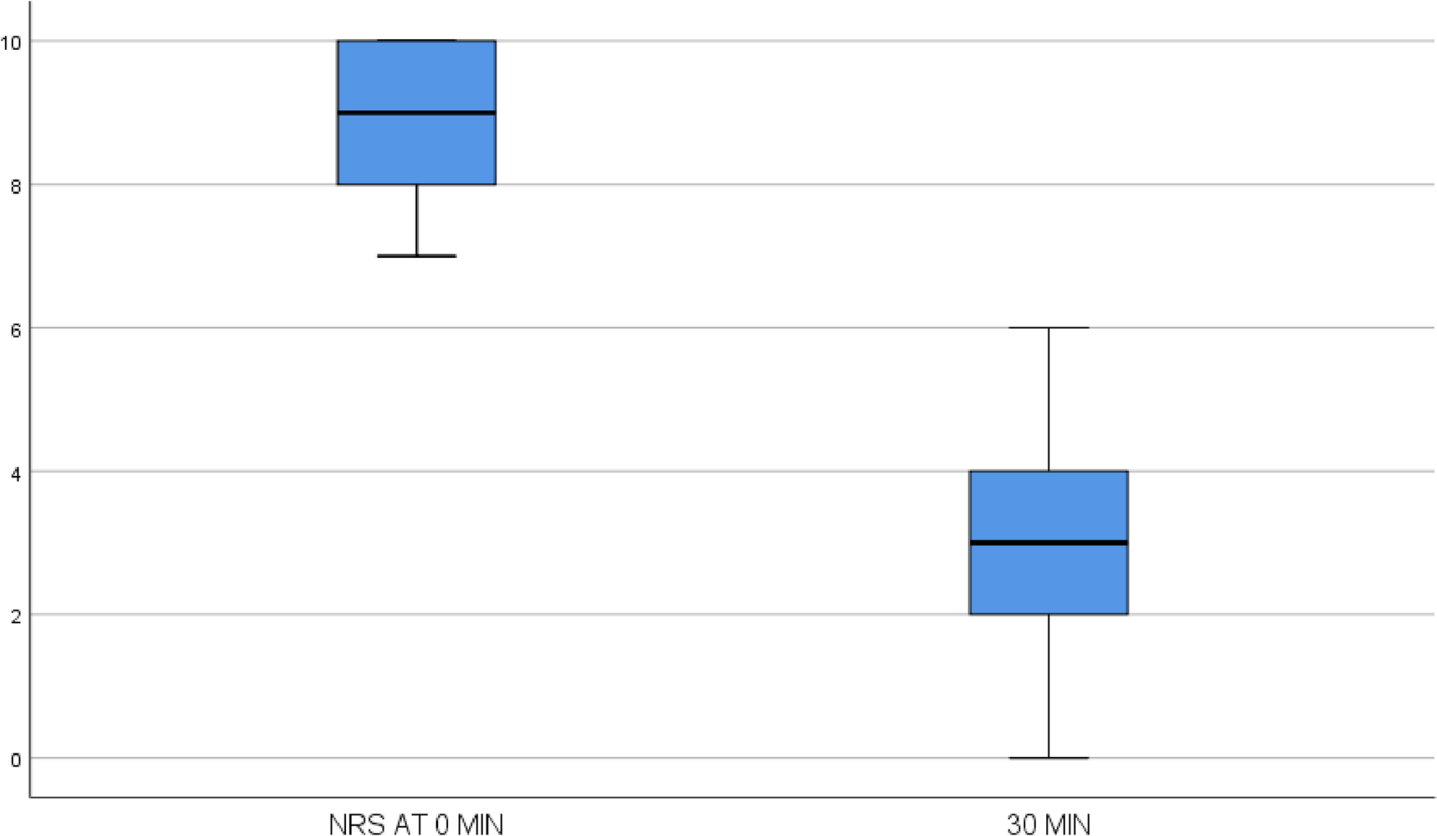
Changes in the NRS score at 30 min

Fentanyl was the most commonly administered analgesic to manage acute pain crisis (47%), followed by morphine (44%). The median NRS pain score decreased from 9 (IQR: 8–10) at baseline to 6 (IQR: 4–7) at 15 min (*p* < 0.01) (Fig. 3) and further to 3 (IQR: 2–4) at 30 min (*p* < 0.01) after analgesic administration (Fig. 4). The mean change in the NRS score was 3 points at 15 min and 6 points at 30 min.

During the study, no adverse events attributable to the use of fentanyl, morphine, or other analgesics were observed or documented among the study participants.

## Discussion

This prospective study provides one of the largest evaluations of acute pain crisis among cancer patients presenting to an outpatient specialist palliative care clinic in India. We found a prevalence of 1.71% (95% CI: 1.38–2.08) in over 5,570 patients screened, a figure that aligns with globally reported rates ranging from 1% to 5%. While relatively infrequent, these episodes represent a significant clinical challenge, constituting a subset of patients requiring urgent symptom control and complex multidisciplinary management. Notably, our findings add to the limited data on this subject in lower- and middle-income countries (LMICs), where structural barriers, late presentation, and resource limitations influence both the prevalence and management of acute pain crises [10]. 

The median patient age was 50 years (IQR: 40–57), reflecting the younger demographic of advanced cancer presentations in the Indian subcontinent than in Western populations. Our cohort was dominated by head and neck cancers (20%), which closely mirrors national cancer epidemiology tied to high rates of tobacco use, followed by gynecological, thoracic, breast, and hepatobiliary malignancies. This pattern is consistent with Snijders et al.’s recent meta-regression, which revealed that the prevalence of pain was highest in gynecological cancers but was also commonly associated with head and neck and gastrointestinal malignancies [4]. The low rate of hematolymphoid malignancies is likely multifactorial, potentially reflecting referral pathways and differing pain burdens in those cancers.

Pain, regarded as the fifth vital sign, exerts a profound toll not only on patients but also on families and caregivers, who experience distress and helplessness as they witness severe, uncontrolled symptoms [11]. Our analysis revealed that most crises were multifactorial in nature: neuropathic descriptors such as radiating, shooting, and electrical pain predominated, highlighting the complexity of pain mechanisms in advanced cancer. These findings underscore the importance of early and accurate assessment of neuropathic features, which require distinct pharmacologic approaches (notably adjuvant use of gabapentinoids and antidepressants), as reflected by the 21% gabapentin and 17% pregabalin use in our population.

Severe pain is frequently accompanied by other symptom clusters, most notably tiredness, anorexia, and a sharp decline in well-being, as captured on the ESAS-r. Not only do these symptoms erode quality of life, but their interplay with psychological distress is well documented. Our study’s median distress score of 8 (IQR: 6–9) reflects a population in acute psychosocial crisis at risk for anxiety and depression—a finding consistent with Ghoshal et al.’s work demonstrating high levels of fatigue and its close association with diminished emotional functioning and quality of life [12]. 

Most patients (67%) were opioid tolerant at presentation, presumably reflecting chronic pain and prior exposure to strong opioids. This led to high mean daily morphine milligram equivalents (MME), averaging 46 mg per day (SD: 36). Opioid-naïve patients (33%) constitute an important subgroup, as they may be more susceptible to opioid-related side effects and require careful titration.

During acute pain crisis, parenteral opioids remain the gold standard for rapid symptom relief—an approach supported by the 2024 National Comprehensive Cancer Network (NCCN) guidelines and 2020 Indian Society for Study of Pain (ISSP) cancer pain management guidelines [13]. In our cohort, intravenous fentanyl (47%) and morphine (44%) were the most commonly administered crisis management agents. Fentanyl, with its rapid onset, hemodynamic stability, and suitability for patients with renal or hepatic impairment, is favored over morphine in certain clinical contexts [14]. Morphine, despite its slower onset (peak effect up to 30 min), remains widely used due to its familiarity, efficacy, and availability [7]. 

Analgesic efficacy in our study was robust, with NRS pain scores declining from 9 at baseline to 6 at 15 min and 3 at 30 min post-administration. This rapid reduction not only surpasses clinically significant thresholds for pain relief but also highlights a timely response to protocols emphasizing immediate-release opioid use and, when appropriate, patient-controlled analgesia.

Notably, 20% of patients were not on any regular oral analgesics prior to acute pain crisis presentation—a finding that could reflect gaps in pain education, access, or adherence, or simply the overwhelming and unexpected nature of acute pain exacerbations.

Our study reinforces existing recommendations from the National Comprehensive Cancer Network (NCCN) and the American Society of Clinical Oncology, which promote a tiered approach: management of background pain and then rapid intervention for breakthrough or crisis episodes via appropriate opioid selection and delivery routes. Fast-acting agents (fentanyl, IV morphine) are recommended for maximum flexibility and rapidity of onset and are tailored to the patient’s organ function and opioid status [6, 7]. 

Adjunctive coanalgesics (paracetamol and gabapentinoids) are widely used, and their effectiveness in reducing opioid requirements and addressing neuropathic pain is supported by current guidelines. Future studies should assess the impact of nonpharmacological modalities, including psychological and spiritual support, which are widely advocated in integrative palliative care models.

Our prevalence of acute pain crisis aligns closely with figures reported by Western and Asian studies focusing on advanced cancer and palliative populations. The multidimensional symptom burden, high opioid requirements, and rapid response to parenteral opioids are consistent with international findings, verifying the generalizability of our results despite local demographic differences [4]. However, direct comparisons of pain quality and management between outpatient and inpatient crisis presentations remain an area for further research.

### Strengths and limitations

This study’s strengths include its large, prospective design, systematic symptom assessment using validated tools (NRS, ESAS-r, Distress Thermometer), and real-world clinical setting. The inclusion of both opioid-naïve and opioid-tolerant patients reflects actual practice and supports generalizability.

However, there are notable limitations. As a single-center study, the findings may not be representative of broader populations, and referral bias cannot be excluded. The absence of long-term follow-up precludes conclusions on pain recurrence, subsequent quality of life, or the incidence of opioid side effects. Documentation of psychological and socioeconomic factors is limited, although these invariably shape acute pain experiences and clinic utilization. Furthermore, the causes of undertreatment or lack of precrisis analgesics in 20% of cases deserve exploration in future research.

### Clinical and research implications

The low but significant prevalence of acute pain crisis urges oncologists and palliative care clinicians to proactively screen for risk factors and maintain quick access to rapid-onset opioid therapy. Frequent reassessment of pain and distress should become routine, using validated scales (such as the ESAS-r, NRS, and Distress Thermometer) to identify crises early and optimize both symptom control and resource use [9].

Our findings also highlight the importance of education—for both patients and families—regarding pain reporting, medication adherence, and crisis self-management. Integrated psycho-oncology and social support must be strengthened in palliative clinics, as distress scores and psychological sequelae are substantial.

Further multicenter studies are needed to explore predictors of pain crisis and evaluate interventions for prevention, rapid triage, and integrated symptom control. Research on barriers to optimal pain management, especially in LMICs, and the role of digital health solutions (telemedicine, e-alerts for pain escalation) will inform future service models.

## Conclusion

Acute pain crisis, although relatively uncommon in outpatient specialist palliative care, represents a burden on advanced cancer patients. Our study demonstrated that rapid, protocol-driven analgesic intervention—most often delivered with intravenous fentanyl or morphine—can achieve prompt and meaningful pain relief. However, persistent high distress and multifactorial symptomatology call for integrated, multidisciplinary strategies that address physical, emotional, and existential suffering. Ongoing research and system-level innovations are needed to further reduce the impact of pain crisis and improve the quality of life for patients with advanced cancer.

## Data Availability

The datasets used and/or analysed during the current study are available from the corresponding author on reasonable request.
